# Supplementary material for: Information Available to Parents Seeking Education about Infant Play, Milestones, and Development from Popular Sources
Source: Behav Sci (Basel). 2023 May 19;13(5):429. doi: 10.3390/bs13050429 (PMC10215939; doi:10.3390/bs13050429)
Supplement: Supplementary file 1 [file behavsci-13-00429-s001.zip › Parent Ed Sources Supp Material S1 Codebook.pdf]

Parent Education Codebook

Definitions:

Play Activity: An item describing something that a person can do with an infant, or something that the infant can do on their own. If multiple activities were described within one sentence, code each activity separately.

Milestone: A behavior that an infant would be expected to perform by a specified time-point.

Toy recommendations: Any object recommended for use by the infant or parent.

Source Information

- Source ID
  - The unique identifier for each source. Created during the search process.
- Primary Coder
  - Initials of the Primary Coder who initially identifies all play activities, milestones, and toy recommendations.
- Secondary Coder
  - Initials of the Secondary Coder who coded all play activities, milestones, and toy recommendations input by the Primary Coder. Any additional activities missed by the Primary Coder are appended to the activities, milestones, or toys identified by the Primary Coder.
- Title
  - Title of the Source, book, or article
  - Year Published
    - Publication Year, if provided. If not, leave blank
  - Date Accessed
    - Date when the PDF or screenshot of the source information was archived for coding
  - Reference Info
    - Citation, link to online source if applicable, and/or link to archived materials.
  - Source Type
    - Select one from the drop-down list:
      - **Book- Commercial Entity:** Book created by a commercial company, such as Pampers or Gymboree, which in addition to providing information, also sells products.
      - **Book- Profession Organization:** Book created by a professional organization, such as the American Academy of Pediatrics
      - **Book- Popular Press:** Book created by a popular press organization, such *Parents magazine*
      - **Book- Individual:** Book created by an individual author

- **Book- Government/Government Agency**: Book created by a government organization or agency, such as the Center for Disease Control and Prevention (CDC)
- **Website- Commercial Entity:** Website, including YouTube videos, created by a commercial company, such as Pampers or Gymboree, which in addition to providing information, also sells products.
- **Website- Professional Organization:** Website, including YouTube videos, created by a professional organization, such as a hospital, Wikipedia, or WebMD
- **Website- Popular Press:** Website, including YouTube videos, created by the popular press, such as *CNN, Today,* or *Parents Magazine*; also select this if the "about me" information or copyright is linked to a "media group"
- **Website- Individual:** Website or blogs, including YouTube videos, created by an individual, as noted in the "about me" information
- **Website- Government/Government Agency**: Website, including YouTube videos, created by a government organization or agency, such as the CDC
- **Apps- Professional Organization:** App created by a professional organization, such as the Bump
- **Apps- Commercial Entity:** App created by a commercial company, such as Pampers or Gymboree, which in addition to providing information, also sells products.
- **Apps- Popular Press:** App created by the popular press, such as *Parents Magazine*
- **Apps- Individual:** App created by an individual
- **Apps- Government/Government Agency:** App created by a government organization or agency, such as the CDC

- Are credentials (i.e., degrees, certifications, licenses earned by the author) provided for the author(s), for example PhD, DPT, MD, PA-C, CRNP, or OT?
  - 0 = No
  - 1 = Yes; copy and paste the credentials into the next field
- Are qualifications provided for the authors(s) or content, for example descriptions of trainings, experience, expertise, background/history (often found in the "about me" or "contact us" pages), sources referenced, or experts quoted?
  - 0 = no
  - 1 = yes; copy and paste the text describing the author's qualifications into the next field
- Are any instructions provided to teach parents how to know if the infant is ready to play (i.e., signs an infant is ready for play or signs of overstimulation)?
  - 0 = No
  - 1 = Yes; copy and paste those instructions into the next field

    - Is information provided describing how development happens (i.e., information about process/theory, factors influencing development)?
        - 0 = No
        - 1 = Yes; copy and paste the text describing how development happens into the next field
    - Is information provided describing the role that parents can play in impacting developmental outcomes for their infants?
        - 0 = No
        - 1 = Yes; copy and paste the text describing the role that parents can play in impacting developmental outcomes for their infants into the next field
    - Presents Milestone Information
        - 0 = No
        - 1 = Yes (If selected, information about each milestone should be coded in the Milestone tab)
    - Presents Play Information
        - 0 = No
        - 1 = Yes (If selected, information about each play activity should be coded in the Play tab)
    - Presents Toy Recommendation Information
        - 0 = No
        - 1 = Yes (If selected, information about each toy recommendation should be coded in the Toy tab)

<u>Play Activities</u>

- Play activity ID
    - The unique identifier for each play activity. Created during the play activity coding process.
- Activity Name
    - Copy and paste the activity name from the source
- Activity Description
    - Copy and paste the full activity description from the source

<u>Milestones</u>

- Milestone ID
    - The unique identifier for each milestone. Created during the milestone coding process.
- Milestone
    - Copy and paste the milestone from the source

<u>Toys</u>

- Toy recommendation ID
    - The unique identifier for each toy recommendation. Created during the toy recommendation coding process.
- Name of recommended toy/object

    - Copy and paste the toy/object name from the source
- Activity Description
    - Copy and paste the activity description from the source
