# Supplementary material for: Information Available to Parents Seeking Education about Infant Play, Milestones, and Development from Popular Sources
Source: Behav Sci (Basel). 2023 May 19;13(5):429. doi: 10.3390/bs13050429 (PMC10215939; doi:10.3390/bs13050429)
Supplement: Supplementary file 1 [file behavsci-13-00429-s001.zip › Parent Ed Sources Supp Material S3 Author Ed Exp Table.pdf]

SUPPLEMENTAL MATERIALS

Table S2: Author Education/Experience Distribution

*The table shows the total number of multiple response selections (n=17 categories) for the Author Education/Experience data (n=137 total responses) including the count within each category and search type, the distribution of author education/experience types within each of the search types (i.e., Play, Milestone, and Development; "% within Search"), the distribution of author education/experience types across each of the search types (i.e., "% within Author Education/Experiences Responses"), and the standardized adjusted residuals (Z) from post-hoc analyses of the Fisher's Exact test evaluating the null hypothesis that all author education/experience responses were similarly distributed among the Play, Milestone, and Development searches. Categories in italics had a total combined frequency among the three searches less than five so were not included in the statistical analyses. * Plus bold font within the table denotes statistical significance below p <0.0027 after Bonferroni adjustments.*

		Author Education/Experience								
		Healthcare	Healthcare, Parent	Parent, Other	Early Childhood Education	Human Services	Parent, Human Services	Parent	Other	*Unspecified*
**Play Search**	**Count**	**13**	10	2	7	1	5	6	4	1
	**% within Search**	**23.21%**	17.86%	3.57%	12.50%	1.79%	8.93%	10.71%	7.14%	1.79%
	**% within Author Education/Experience Responses**	**24.07%**	66.67%	18.18%	70.00%	11.11%	71.43%	85.71%	57.14%	33.33%
	**Adjusted Residual**	**-3.23***	2.15	-1.60	1.95	-1.88	1.69	2.48	.90	--
**Milestone Search**	**Count**	22	3	6	1	4	0	1	1	1
	**% within Search**	52.38%	7.14%	14.29%	2.38%	9.52%	0%	2.38%	2.38%	2.38%
	**% within Author Education/Experience Responses**	40.74%	20.00%	54.55%	10.00%	44.44%	0%	14.29%	14.29%	33.33%
	**Adjusted Residual**	2.06	-.95	1.79	-1.47	.93	-1.81	-.96	-.96	--
**Development Search**	**Count**	19	2	3	2	4	2	0	2	1
	**% within Search**	48.72%	5.13%	7.69%	5.13%	10.26%	5.13%	0%	5.13%	2.56%
	**% within Author Education/Experience Responses**	35.19%	13.33%	27.27%	20.00%	44.44%	28.57%	0%	28.57%	33.33%
	**Adjusted Residual**	1.41	-1.38	-.09	-.62	1.10	.01	-1.71	.01	--
**Total**	**Count**	54	15	11	10	9	7	7	7	3
	**% within Search**	39.42%	10.95%	8.03%	7.30%	6.57%	5.11%	5.11%	5.11%	2.19%
	**% within Author Education/Experience Responses**	100.00%	100.00%	100.00%	100.00%	100.00%	100.00%	100.00%	100.00%	100.00%

		Author Education/Experience								Total
		Healthcare, Parent, Early Childhood Education	*Healthcare, Other*	*Parent, Early Childhood Education*	*Parent, Early Childhood Education, Other*	*Healthcare, Early Childhood Education*	*Early Childhood Education, Parent*	*Healthcare, Human Services*	*Parent, Early Childhood Education, Human Services*	
**Play Search**	**Count**	0	0	2	2	1	1	0	1	56
	**% within Search**	0%	0%	3.57%	3.57%	1.79%	1.79%	0%	1.79%	100.00%
	**% within Author Education/Experience Responses**	0%	0%	100.00%	100.00%	50.00%	100.00%	0%	100.00%	40.88%
	**Adjusted Residual**	--	--	--	--	--	--	--	--	N/A
**Milestones Search**	**Count**	1	1	0	0	0	0	1	0	42
	**% within Search**	2.38%	2.38%	0%	0%	0%	0%	2.38%	0%	100.00%
	**% within Author Education/Experience Responses**	33.33%	50.00%	0%	0%	0%	0%	100.00%	0%	30.66%
	**Adjusted Residual**	--	--	--	--	--	--	--	--	N/A
**Development Search**	**Count**	2	1	0	0	1	0	0	0	39
	**% within Search**	5.13%	2.56%	0%	0%	2.56%	0%	0%	0%	100.00%
	**% within Author Education/Experience Responses**	66.67%	50.00%	0%	0%	50.00%	0%	0%	0%	28.47%
	**Adjusted Residual**	--	--	--	--	--	--	--	--	N/A
**Total**	**Count**	3	2	2	2	2	1	1	1	137
	**% within Search**	2.19%	1.46%	1.46%	1.46%	1.46%	0.73%	0.73%	0.73%	100.00%

	**% within Author Education/Experience Responses**	100.00%	100.00%	100.00%	100.00%	100.00%	100.00%	100.00%	100.00%	100.00
